# Validity and reliability of Household Disinfectants-Cleaners Questionnaire (HDCQ) to investigate public awareness and performance in the Emirate of Abu Dhabi

**DOI:** 10.1186/s12889-025-22317-y

**Published:** 2025-03-29

**Authors:** Mona Aridi, Elgenaid Hamadain, Wissam Ghach, Nisreen Alwan

**Affiliations:** 1 LARIS, SFR Mathstic, University of Angers, 49000 Angers, France; 2 College of Health Sciences, Abu Dhabi University, Abu Dhabi, United Arab Emirates; 3 Department of Public Health, Canadian University Dubai, Dubai, United Arab Emirates; 4 The Global Health Network-Middle East and North Africa (TGHN-MENA), Dubai, United Arab Emirates

**Keywords:** Questionnaire validation, Public awareness, Household disinfectants, Household cleaners, Safe practices

## Abstract

**Background:**

Although household disinfectant cleaners are used at home frequently, less research has focused on standardized assessment tools to measure public awareness and precautions in practice, especially during health crises. The COVID-19 pandemic highlighted the important role of household disinfectants-cleaners in preventing infections. However, poor public awareness and unsafe practices can undermine their effectiveness. This study indirectly addresses this lack by developing and validating the Household Disinfectants-Cleaners Questionnaire (HDCQ), to make available an evidence-based comprehensive tool to support future assessments, public health research, and policy development.

**Objective:**

To assess the validity and reliability of a questionnaire designed to measure public awareness and performance regarding the safe use of household disinfectants-cleaners in Abu Dhabi.

**Methods:**

A cross-sectional Google survey, involving a sample of 750 Abu Dhabi residents, was analyzed using IBM-SPSS (Version 27.0) and RStudio (Version 1.1.456, Inc., 2009–2018). Validity tests included face, content, and construct evaluations by nine academic experts based on seven parameters. Reliability was assessed through Cronbach’s alpha and inter-item correlation. Principal Factor Analysis (PFA), including the Kaiser–Meyer–Olkin (KMO) measure and Bartlett’s test, was conducted for each section in SPSS. Exploratory Factor Analysis (EFA) and Confirmatory Factor Analysis (CFA) in RStudio identified the underlying factors explaining item covariation.

**Results:**

Cronbach’s alpha values for the awareness and performance domains were 0.854 and 0.883, indicating high internal consistency. Expert ratings indicated high face validity, with importance levels ranging from 0.89 to 1.00. The average Content Validity Ratio (CVR) for the questionnaire was 0.95, indicating strong content relevance. The PFA results demonstrated adequate sampling adequacy, with a KMO measure values of 0.879 and 0.891 and Bartlett’s test was statistically significant (χ2 = 2,368, *P* < 0.001) and (χ2 = 3,274, *P* < 0.001) for each section respectively, confirming factorability and suitability for factor analysis. Both EFA and CFA indicated a good model fit with RMSEA of 0.077 for Sect. 1 and 0.090 for Sect. 2, CFI of 0.990 for Sect. 1 and 0.975 for Sect. 2, and TLI of 0.986 for Sect. 1 and 0.967 for Sect. 2, confirming the robustness of the factor structure and the questionnaire’s validity.

**Conclusions:**

The findings demonstrate that this questionnaire is a valid and reliable tool for assessing public awareness and performance regarding the safe use of household disinfectants-cleaners for future pandemics. Recognizing inherent limitations, detailed in the conclusion section, is essential for effective application in public health contexts.

**Supplementary Information:**

The online version contains supplementary material available at 10.1186/s12889-025-22317-y.

## Background

Amidst the persistent challenges posed by the Coronavirus Disease 2019 (COVID-19), the importance of public awareness and commitment to safe use of household disinfectants-cleaners are critical factors in curbing the viral transmission. Therefore, health organizations and public health researchers worldwide are implementing strategies to lower transmission rates, gaining valuable insights into the public's knowledge and behaviors concerning the use of disinfectants and cleaners, which has become a crucial necessity [1–5].

Since the onset of the pandemic, many incidents of accidental poisoning worldwide linked to the improper use of disinfectants-cleaners have been reported [6–8]. Between January and March 2020, 45,550 exposure-related calls concerning cleaners (28,158) and disinfectants (17,392) were reported by poison centers. Such reportsmarked a rise of 20.4% compared to the same period in 2019 (37,822 calls) and 16.4% compared to 2018 (39,122 calls) [9]. This has led to rising concerns about contracting Severe Acute Respiratory Syndrome Coronavirus-2** (**SARS-CoV-2) which prompted the public community to intensify their hygiene practices by increasing disinfectant usage and inadvertently elevating the risk of chemical exposure when misused [10–13]. Data from the Dammam Poison Control Center in Saudi Arabia revealed a notable rise in public exposure to surface disinfectants and hand sanitizers between 2019 and 2020 [14]. Moreover, the Centers for Disease Control and Prevention (CDC) documented a significant surge in chemical exposures, including COVID-related cleaners and disinfectants, in the United States, as reported in the Morbidity and Mortality Weekly Report (April 2020) [9]. Investigations revealed a dearth of knowledge and subsequently risky practices concerning the safe handling of household cleaning and disinfection products during the COVID-19 outbreak in the United States [15]. Literature suggests that public awareness, practices, and information sources are interconnected variables influencing the safe use of chemical disinfectants among populations [12, 13, 15–17].

In the UAE, a noticeable surge in awareness among individuals has been observed, attributed to the organization of campaigns and pandemic-related activities across the internet and social media platforms [18, 19]. Only one study by Alwan et al. (2023) examined the public community's awareness and performance concerning the safe use of cleaners and disinfectants at the household level in the Emirate of Abu Dhabi, UAE providing new insights into public awareness regarding the proper handling of household chemicals and their potential implication for community health, in alignment with the Sustainable Development Goal 3 (SDG3) [20]. A similar study was conducted in Lebanon to highlight the poor awareness and misuse of household cleaning products and disinfectants during the spread of COVID-19 [13]. However, there are no other similar studies in the Middle Eastern countries where a notable rise in public exposure to surface disinfectants and hand sanitizers were noticed.

The COVID-19 pandemic has led to a huge rise in survey-based research [21, 22]. Findings from previous research indicated limited knowledge and high-risk practices with respect to safe handling of chemical-based disinfectants and cleaning products during the spread of COVID-19 [12, 13, 16, 20, 23]. A May 2020 online survey found significant knowledge gaps in household cleaners’ and disinfectants’ safety, with one-third of respondents engaging in hazardous practices like washing food with bleach or inhaling these products [17]. Unfortunately, the questionnaires used in most of the published studies mentioned above were either not validated [17] or were partially validated [23] using a panel of experts or content validation. Recently, a new assessment tool was validated to evaluate the public levels of awareness and performance regarding the safe use of chemical-based disinfectants [24]. However, this tool was limited to the use of chemical-based disinfectants (mostly hand and object sanitizers) on the personal level. Therefore, it is pivotal to validate questionnaires designed to assess public awareness and performance towards the safe use of household disinfectant-cleaners where harmful cleaning products are utilized to clean contaminated surfaces and where vulnerable people (*e.g.* children, elderly people, and other immunocompromised patients) are expected to be highly exposed to these harmful chemicals. This ensures that they effectively measure the intended constructs, providing reliable data essential for informing targeted interventions, policy decisions, and public health strategies for future pandemics.

According to the literature [25, 26] Principal Factor Analysis (PFA), Exploratory Factor Analysis (EFA), and Confirmatory Factor Analysis (CFA) are used to strictly assess the proper household practices. PFA addresses data complexity by identifying the underlying constructions running domestic behavior. EFA examines potential relations between survey goods without implementing pre-defined beliefs, when specific dimensions of awareness and practices are not fully understood. The CFA strictly tests the hypothesis about the structure of the equipment, ensuring that it aligns with theoretical models [27]. Employing these methods will provide a more sophisticated understanding of the dimensions of domestic sanitation disinfections and of practices, providing a piece of valid tool for future research and intervention and a method for rigorous means of verification [28].

The novelty of this study lies in its focus on validating a specialized tool for assessing public awareness and practices regarding the safe use of household disinfectants. While many studies have evaluated general knowledge and behaviors related to infection control, few have developed and validated instruments specifically addressing disinfectant use. This paper's novelty is further underscored by its rigorous validation framework, encompassing face, content, and construct validity, along with advanced statistical methods, including Principal Factor Analysis (PFA), Exploratory Factor Analysis (EFA), and Confirmatory Factor Analysis (CFA). Additionally, by adapting the questionnaire to the specific socio-cultural context of Abu Dhabi, this study provides an adaptable instrument that can inform more precise public health interventions in diverse populations, making a meaningful contribution to the field of public health research.

The aim of this study is to rigorously evaluate the validity and reliability of the Household Disinfectants-Cleaners Questionnaire (HDCQ) tool which is developed to measure public awareness and performance concerning the safe use of household disinfectants as a preventive measure against COVID-19 in the emirate of Abu Dhabi. This validation process focuses on several parameters, including difficulty, obscurity, importance, simplicity, clarity, appropriateness, and relevance, to ensure that the tool is accurate, consistent, and applicable across various demographic contexts within Abu Dhabi.

## Methods

A comprehensive validation process was undertaken to ensure the effectiveness and relevance of the HDCQ. This process included initial consultations for face and content validity with a panel of nine experts, followed by a pilot study involving a sample of 750 respondents (Youngsters aged less than 18 years old were excluded from this study) to evaluate construct validity. Participants were recruited using an online convenience sampling method, where the survey was distributed across various digital platforms to reach a broad audience. The following sections outline the specific methodologies employed in these validation steps.

### Study instrument

The questionnaire to be validated was adapted from the Morbidity and Mortality Weekly Report, 2020, Centers for Disease Control and Prevention [9]. The HDCQ questionnaire consisted of 18 items divided into two domains: awareness and performance. There were nine items in the awareness domain with a 4-point Likert scale (Strongly agree, somewhat agree, somewhat disagree, and strongly disagree) and nine items in the performance domain with seven items of a 3-point Likert scale (Always, sometimes, never) and two binary items (Yes/no questions).

The HDCQ questionnaire was emailed to nine expert academicians in the fields of public health, chemistry, biology, and environmental health for review. This multidisciplinary selection was intended to ensure a comprehensive evaluation of the questionnaire’s content validity. The experts were requested to give their opinion (ranking) on seven parameters for each domain (awareness and performance) using a 4-point Likert scale. The seven questionnaire parameters contribute to the face validity and content validity qualitatively and quantitatively including the test of difficulty (1 = very difficult, 4 = not difficult), obscurity (1 = very obscure, 4 = not obscure), level of importance (1 = not important, 4 = very important), simplicity (1 = not simple, 4 = very simple), clarity (1 = not clear, 4 = very clear), test of appropriateness (1 = minimum, 4 = maximum), and level of relevance (1 = not relevant, 4 = very relevant). Those parameters assess the items qualitatively and quantitatively through ratings and calculated ratios. The expert panel determined whether the contents of the questionnaire were relevant to the conceptual framework. They also determined whether the number of items in each domain was sufficient to represent the questionnaire.

The study protocol was approved by the Institutional Review Board (IRB) at Abu Dhabi University (CoHS-21–10–32) in accordance with the Declaration of Helsinki. The questionnaire comprised a consent form that familiarized respondents (equal or above the age of 18 years) with the study's objectives, advantages, confidentiality measures, and the absence of foreseeable risks. Furthermore, it reiterated that participation in the study was voluntary, and completing the questionnaire signified the individual's agreement to participate.

### Face validity

To assess the appropriateness of an indicator as a valid measure of its underlying construct based on its apparent suitability, face validity was established. It was computed by dividing the sum of the rankings of the nine experts by the sum of the maximum rankings (36) based on Salkind (2010) [29]. Face validity was established through the tests of difficulty, obscurity, and the level of importance for the two domains.

### Content validity

Content validity was defined by Bollen in 2014 [30] as a qualitative form of validity that evaluates whether the expressions contained in the measuring instrument represent the phenomenon intended to be measured. Therefore, content validity reveals the extent to which each item in the questionnaire serves the purpose. Several methods have been proposed to determine content validity by taking expert opinions. This transforms qualitative studies into quantitative ones [31]. To obtain objective results in the calculation of content validity, the quality and number of experts have significant importance as pointed out by Ayre and Scally in 2014 [32]. Qualified experts are crucial for consistency and unbiased results, and academicians or practitioners with great knowledge are preferred. Lawshe’s model [33] was adopted to perform a quantitative evaluation of the items by computing the Content Validity Ratio (CVR) using the following formula:

$${\mathrm{CVR}}_{\mathrm i}=\frac{{\mathrm N}_{\mathrm e}-{\displaystyle\frac{\mathrm N}2}}{\displaystyle\frac{\mathrm N}2}$$

where CVR_i_ is the CVR value for the i^th^ measurement item, N_e_ is the number of experts indicating strong agreement, and N is the total number of experts. The content validity was achieved qualitatively through its three testing parameters: the test of simplicity, the test of clarity, and the test of appropriateness, and quantitatively through the level of relevance test.

Furthermore, the Item level Content Validity Index (I-CVI) was determined to measure the level of agreement among experts. The Universal Agreement (UA) was calculated by dividing the number of items with a CVI of scoring 1.00 by the total number of items analyzed, resulting in Scale Content Validity Index S-CVI/UA. The average also was calculated by dividing the total sum of CVIs by 18.

### Construct validity

**Fig. 1:**
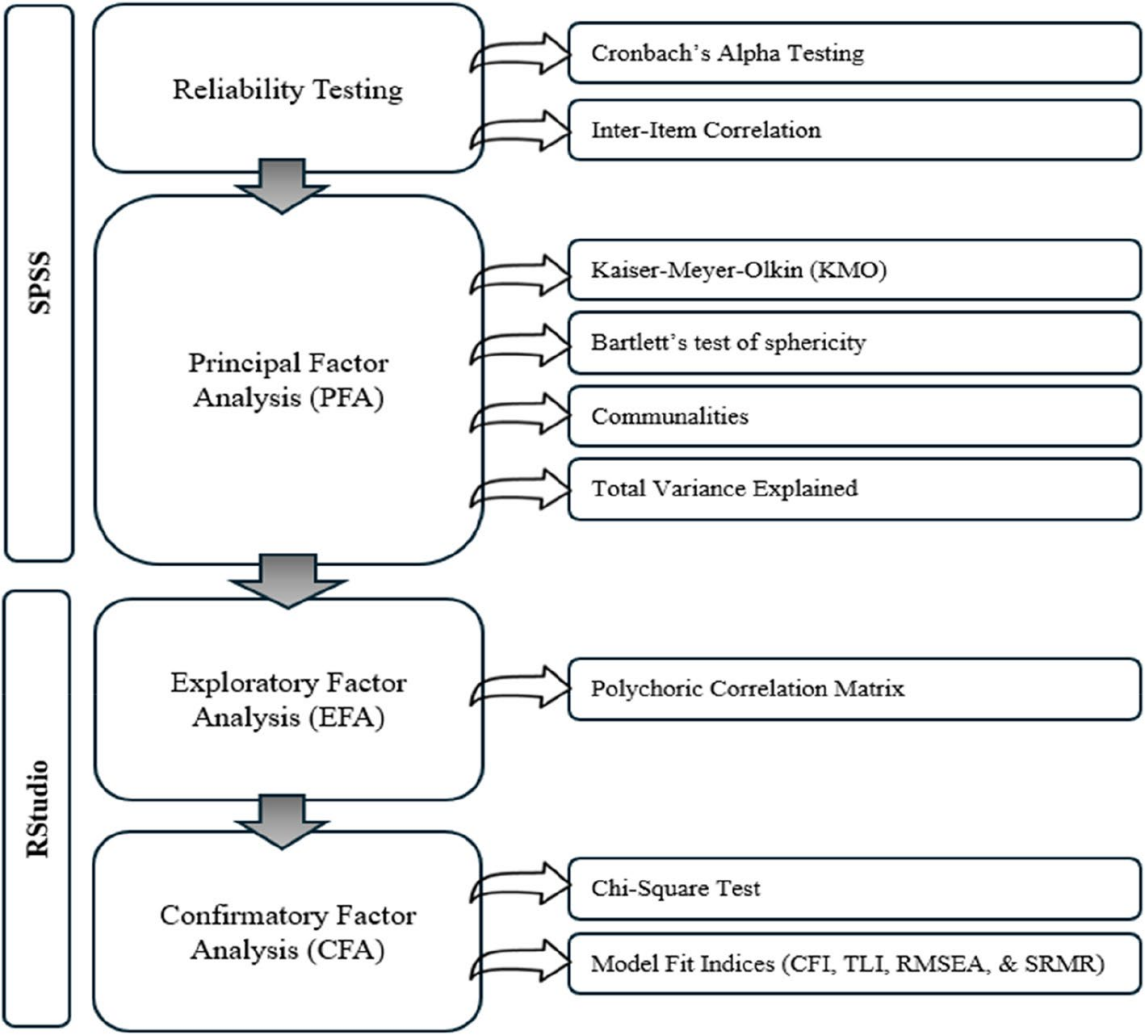
Outline of the construct validity process

To establish the construct validity, a pilot study aimed at confirming that the questionnaire items accurately represented the theoretical constructs of awareness and performances related to household cleaning disinfectants. Construct validity was evaluated through statistical analysis responses, ensuring that the items grouped together as expected based on their conceptual relevance. This stage involved a series of principal, exploratory, and confirmatory factor analyses to identify underlying factor structures within each section of the questionnaire. The outline of the construct validity steps is presented in Fig. 1.

The revised questionnaire was a pilot one tested with a sample of 750 participants to assess construct validity. This pilot testing provided an initial evaluation of the questionnaire’s capacity to measure the intended constructs accurately, using SPSS and RStudio.

#### Reliability analysis

To evaluate the internal consistency, Cronbach’s alpha was computed separately for each section of the questionnaire using IBM Statistical Package for Social Sciences (SPSS, Version 28). Additionally, an inter-item correlation analysis was conducted to ensure that the items within each section measured the same underlying construct also using SPSS. To maintain consistency, reverse coding was applied to negatively worded items: in Sect. 1 (Awareness), items K7 and K8 were reverse-coded and labeled as K7_rev and K8_rev, while in Sect. 2 (Performance), item P5 was reverse-coded and labeled as P5_rev. This approach ensured that all items contributed appropriately to the assessment of their respective constructs.

#### Principal Factor Analysis (PFA)

After assessing the reliability through Cronbach’s alpha for internal consistency and inter-item correlations to verify that each section’s items measured the same underlying construct, PFA was conducted in SPSS to explore dimensionality. According to Taherdoost et al. (2014) [34], sampling adequacy was confirmed by the Kaiser–Meyer–Olkin (KMO) measure, with a cut-off value of 0.6 [35], and Bartlett’s test of sphericity, with statistical significance at *P* < 0.05 indicating that factor analysis was appropriate. Communalities were evaluated, and items with communalities above 0.3 were considered suitable for retention [36], supporting the item structure for subsequent analysis. Both the reliability analysis and PFA were performed using IBM-SPSS.

#### Exploratory Factor Analysis (EFA)

Following the PFA, EFA was subsequently conducted in RStudio (Version 1.1.456, Inc., 2009–2018) to further explore the latent structure within each section. Polychoric correlations were calculated for the ordinal items in each section, accounting for the ordinal scale used and enhancing the accuracy of item correlations. Factor loadings were calculated to evaluate how strongly each item was associated with a particular factor (ML1 and ML2) using a cutoff value of 0.4 for comparison (Values should be greater than 0.4) [37, 38]. For Sect. 2, which contained two binary items, tetrachoric correlation was deemed unsuitable due to the limited item count, hence it may not perform optimally with a limited number of items, leading to unstable estimates [39]. Instead, item-total correlations were calculated to assess the relationship between each item and the overall score, providing a straightforward measure of item consistency [40]. This approach confirmed factor loadings in each section and indicated that items were unidimensional, thereby reinforcing construct validity.

#### Confirmatory Factor Analysis (CFA)

To validate the factor structure identified during the EFA, CFA was performed for each section in RStudio. The Diagonally Weighted Least Squares (DWLS) estimator was chosen for its suitability with ordinal data. The optimization process was carried out using the Non-Linear MINimization using Bounds (NLMIN)B method, which efficiently handles parameter estimation and maximizes the likelihood function. This approach ensures robust and reliable factor loadings for assessing the underlying structure of the awareness section.

Model fit was assessed through several indices: the Comparative Fit Index (CFI) and Tucker-Lewis Index (TLI), with values above 0.90 indicating a good fit, as well as the Chi-Square test and corresponding *P*-value. Additionally, the Root Mean Square Error of Approximation (RMSEA) and Standardized Root Mean Square Residual (SRMR) were examined, with acceptable cut-off values of less than 0.08. Latent variable correlations and factor loadings were also reviewed to confirm alignment between the observed data and the hypothesized structure.

## Results

The findings from HDCQ provide critical insights into public knowledge and behaviors related to the safe use of household disinfectants-cleaners. The data collected from the nine experts and the pilot study highlight key trends and patterns in awareness levels and practices among respondents. This section presents the results of the validation process, including descriptive statistics and statistical analyses, to illustrate the reliability and applicability of the questionnaire in assessing chemical awareness and practices in household settings.

### Face validity

Examining the outcomes of the two qualitative criteria for face validity, specifically, the difficulty and clarity of each item, the ratings provided by the nine experts varied from a minimum of 0.89 to a maximum of 1.00 (Table 1).

**Table 1:** Face validation of the nine items for the two domains: awareness and performance

Qualitative Assessment						Quantitative Assessment		
**Difficulty**	AWR^a^	PERF^b^	**Obscurity**	AWR^a^	PERF^b^	**Level of Importance**	AWR^a^	PERF^b^
(1)	1.00	1.00	(1)	1.00	0.97	(1)	0.97	1.00
(2)	1.00	1.00	(2)	1.00	1.00	(2)	0.97	0.97
(3)	1.00	1.00	(3)	1.00	1.00	(3)	1.00	0.94
(4)	1.00	1.00	(4)	1.00	1.00	(4)	0.97	1.00
(5)	1.00	0.97	(5)	1.00	0.94	(5)	1.00	0.97
(6)	1.00	0.97	(6)	1.00	0.89	(6)	0.97	0.94
(7)	0.89	0.97	(7)	0.97	0.89	(7)	0.94	0.94
(8)	0.92	0.97	(8)	0.94	0.89	(8)	0.94	0.94
(9)	0.92	0.97	(9)	0.97	0.89	(9)	0.92	0.94

^a^AWR stands for awareness

^b^PERF stands for performance

Beginning with the difficulty ratings in the qualitative assessment for each item, the lowest level of agreement was 89% for item 7 of awareness. For obscurity, items 6, 7, 8, and 9 also received the lowest agreement of 89%. Quantitatively, the minimum agreement attained at the level of importance was for item 9 of awareness. Both assessments indicate that while most items demonstrate a high level of clarity, relevance, and simplicity, items 6 through 9 in the awareness section received slightly lower agreement scores. However, these items still meet acceptable thresholds, suggesting that all items are suitable for inclusion without further modification.

### Content validity

According to the qualitative evaluation of the content represented by testing the simplicity, clarity, and appropriateness of the items, the lowest level of agreement was noticed for the last item of performance with a share of 86%. For the clarity and the simplicity criteria, none of the items received an agreement below 94%, reflecting a quite acceptable assessment (Table 2).

**Table 2:** Content validity of the nine items for the two domains: awareness and performance

**Qualitative Assessment**									
**Simplicity**	AWR^a^	PERF^b^		**Clarity**	AWR^a^	PERF^b^	**Appropriateness**	AWR^a^	PERF^b^
(1)	1.00	1.00		(1)	1.00	1.00	(1)	0.97	1.00
(2)	1.00	1.00		(2)	1.00	1.00	(2)	0.97	1.00
(3)	1.00	1.00		(3)	1.00	1.00	(3)	1.00	1.00
(4)	1.00	1.00		(4)	1.00	1.00	(4)	1.00	1.00
(5)	1.00	0.94		(5)	1.00	0.94	(5)	1.00	0.94
(6)	1.00	0.94		(6)	1.00	0.94	(6)	1.00	0.89
(7)	0.89	0.94		(7)	0.97	0.94	(7)	0.97	0.89
(8)	0.89	0.94		(8)	0.97	0.94	(8)	0.97	0.86
(9)	0.92	0.94		(9)	0.97	0.94	(9)	0.0.97	0.86
**Quantitative Assessment (CVR according to Lawshe’s model)**									
			**Level of Relevance**		AWR^a^	PERF^b^			
			(1)		1.00	1.00			
			(2)		1.00	1.00			
			(3)		1.00	1.00			
			(4)		1.00	1.00			
			(5)		1.00	1.00			
			(6)		1.00	0.78			
			(7)		1.00	0.78			
			(8)		1.00	0.78			
			(9)		1.00	0.78			

^a^AWR stands for awareness

^b^PERF stands for performance

#### Content validity ratio

For the quantitative assessment, the level of relevance was evaluated numerically using the Content Validity Ratio (CVR). Based on Laswhe’s model, any item rated by nine experts with a CVR < 0.78 was considered non-essential and recommended for removal. However, none of the 18 items fell below the critical value of 0.78, so none of the items needed to be removed. Furthermore, the average CVR for the entire questionnaire was 0.95, indicating a highly acceptable level. The (CVR) critical value for a panel size of 9 is 0.78.

#### Content validity index

In Table 2, the I-CVI ranged between 0.88 and 1.00, indicating a high level of agreement among experts. The UA was calculated by dividing the number of items with a CVI of 1.00 (14 items) by the total number of items analyzed (18 items), resulting in Scale Content Validity Index S-CVI/UA = 0.78. The average was calculated by dividing the total sum of CVIs by 18, giving S-CVI/Ave = 0.98. Both the UA and average methods demonstrated high content validity, with S-CVI/UA > 0.75 and S-CVI/Ave > 0.90.

### Construct validity

To examine construct validity, a set of consecutive analyses were applied on awareness and performance domains, starting with PFA followed by EFA, and finally validated by CFA. This is a data reduction technique that uses mathematical procedures to simplify intercorrelated measurements to explore patterns in a set of variables. It groups together questions that are highly correlated to extract a smaller set of components that retain a high proportion of the information in the original questions. Factor analysis allows irrelevant questions to be removed from the final questionnaire and allows extracting valuable components from the data set of a questionnaire survey. This provides very valuable input for the decision-makers to focus on few important factors rather than a large number of variables [41].

Convenience sampling was applied to collect a total of 750 participants in this pilot study and validate the HDCQ tool. The sample was almost evenly split by gender, with slightly more females (52.9%) than males. Most participants held a bachelor’s degree (60.5%), while smaller percentages had lower or higher educational qualifications. Age distribution showed a younger demographic, with over three-quarters (75.3%) aged between 15 and 30, with a mean age of 27.68 for the entire sample ranging between a minimum of 18 and a maximum of 70. Participants were drawn from three main provinces, with representation across Abu Dhabi (35.2%), Al-Dhafra (28.0%), and Al-Ain (36.8%). Additionally, 45.7% of respondents reported having contracted COVID-19, and 57.2% indicated prior contact with a COVID-19 patient. This diverse sample provided a comprehensive basis for validating the questionnaire’s construct reliability (Table 3.).

**Table 3:** Demographic characteristics of the pilot study participants

		**Frequency**	**Percentage (%)**
Gender	Males	353	47.1
	Females	397	52.9
Educational Level	No Education	25	3.3
	High School Junior	89	11.9
	Baccalaureate	97	12.9
	Bachelor	454	60.5
	Master	63	8.4
	Doctoral	22	2.9
Province	Abu Dhabi	264	35.2
	Al-Dhafra	210	28.0
	Al-Ain	276	36.8
Age	15 - 30	565	75.3
	31 - 45	152	20.3
	46 - 60	33	4.4
Have you been infected earlier with COVID-19?	Yes	343	45.7
	No	407	54.3
Have you been in contact with a COVID-19 patient?	Yes	429	57.2
	No	321	42.8

#### Reliability testing

In the reliability analysis, Cronbach’s alpha was calculated for each section to assess internal consistency. For Sect. 1 (Awareness), Cronbach’s alpha was 0.854, indicating good reliability. For Sect. 2 (Performance), Cronbach’s alpha was 0.883, suggesting high reliability. Both values exceeded the commonly accepted threshold of 0.70, supporting the internal consistency of the items in each section (Table 4).

**Table 4:** Cronbach's alpha values for each section

	Awareness	Performance
Cronbach’s alpha	0.854	0.883
Number of items	9	9

The inter-item correlation matrix for the awareness section (Table S1, Appendix A) and performance section (Table S2, Appendix A) shows the relationships between items within each section.

Most inter-item correlations for each of the awareness items (Table S1, Appendix A) and performance items (Table S2, Appendix A), fall between 0.3 and 0.6, which suggests moderate correlations. This range typically indicates that the items are related but not redundant, which is desirable for capturing distinct yet related aspects of awareness and performance in household disinfectant practices.

The range of correlations supports the internal consistency of each of the awareness and performance items, aligning with the Cronbach’s alpha values obtained. These matrices justify that the items in each section collectively measure a unified construct while allowing for variation among specific behaviors.

#### Principal Factor Analysis (Awareness)

##### KMO and Bartlett's Test

The KMO measure of sampling adequacy was 0.879, which is well above the recommended threshold of 0.6, indicating that the data is suitable for factor analysis. Bartlett’s test of sphericity was significant (χ^2^ = 2,368.099, degrees of freedom (df) = 36, *P* < 0.001), confirming that the correlation matrix is not an identity matrix, and therefore, the variables are interrelated and suitable for factor analysis (Table 5).

**Table 5:** KMO and Bartlett's tests for items of awareness

KMO Measure of Sampling Adequacy		0.879
Bartlett's Test of Sphericity	Approx. Chi-Square	2,368.099
	df	36
	Sig.	.000

##### Communalities (Extraction Method: Principal Component Analysis)

The communalities (Table 6) indicate the proportion of each item’s variance explained by the extracted factors. Most items show moderate to high communalities, with values ranging from 0.352 (A2) to 0.564 (A1), suggesting that these items share a fair amount of variance with the underlying factors.

**Table 6:** Communalities for the items of awareness

Communalities (Extraction Method: Principal Component Analysis)					
A1	0.564	A4	0.538	A7_rev	0.418
A2	0.352	A5	0.505	A8_rev	0.410
A3	0.516	A6	0.468	A9	0.495

##### Total variance explained

In the total variance explained results presented in Table S3 (Appendix A), the first component has an eigenvalue of 4.266, explaining 47.4% of the variance, indicating it is the most influential factor in this section. The second component has an eigenvalue of 0.980, adding 10.9% to the explained variance, for a cumulative total of 58.3%. Components beyond the second explain less than 10% of the variance each and are therefore less significant.

#### Principal Factor Analysis (Performance)

##### KMO and Bartlett's Test

The KMO measure of sampling adequacy was 0.891, significantly exceeding the recommended minimum of 0.6, which indicates that the data is appropriate for factor analysis. Bartlett’s test of sphericity was also significant (χ^2^ = 3,274.020, df = 36, *P* < 0.001), confirming that the correlation matrix is not an identity matrix. This result suggests the variables are sufficiently correlated for factor analysis to be applicable (Table 7).

**Table 7:** KMO and Bartlett's tests for items of performance

KMO Measure of Sampling Adequacy		0.891
Bartlett's Test of Sphericity	Approx. Chi-Square	3,274.020
	df	36
	Sig.	.000

##### Communalities (Extraction Method: Principal Component Analysis)

The communalities (Table 8) reflect the proportion of each item accounted for by the extracted factors. Most items indicate moderate to high communalities, ranging from 0.476 (P5_rev) to 0.703 (P9), suggesting that these items have a substantial amount of shared variance with the underlying factors.

**Table 8:** Communalities for items of performance

Communalities (Extraction Method: Principal Component Analysis)					
P1	0.554	P4	0.507	P7	0.504
P2	0.544	P5_rev	0.476	P8	0.570
P3	0.524	P6	0.504	P9	0.703

##### Total variance explained

In the total variance explained outcomes displayed in Table S4 (Appendix A), the first component has an eigenvalue of 4.884 and accounts for 48.8% of the variance, making it the most significant factor in this section. The second component has an eigenvalue of 0.942, contributing an additional 10.46% to the explained variance, bringing the cumulative total to 64.73%. Subsequent components each explain less than 10% of the variance and are thus less impactful.

#### Exploratory Factor Analysis (Awareness)

For the first section, EFA was conducted using a polychoric correlation matrix with varimax rotation (fm = ‘m1’) in RStudio to identify the underlying structure of the data (Table 9). Each item (A1 to A9) has loadings on two factors (Labeled ML1 and ML2). Factor 1 (ML2) explains 3.69 of the total variances, while Factor 2 (ML1) explains 2.15. ML2 accounts for 41% of the total variance, and ML1 accounts for 24%, and together they explain 65% of the variance in the dataset.

**Table 9:** Polychoric correlation results for the items of awareness

		**ML2**	**ML1**
SS Loadings		3.69	2.15
Proportion Variance		0.41	0.24
Cumulative Variance		0.41	0.62
Proportion Explained		0.63	0.37
Cumulative Proportion		0.63	1.00
Correlation of (Regression) Scores with Factors		0.94	0.99
Mean Item Complexity			1.5
Model Fit Statistics $$\left\{\begin{array}{l}\mathrm{Root}\;\mathrm{Mean}\;\mathrm{Square}\;\mathrm{of}\;\mathrm{the}\;\mathrm{Residuals}\;\left(\mathrm{RMSR}\right)\\\mathrm{Corrected}\;\mathrm{RMSR}\\\mathrm{Fit}\;\mathrm{based}\;\mathrm{upon}\;\mathrm{Off}-\mathrm{Diagonal}\;\mathrm{Values}\end{array}\right.$$			0.04
			0.05
			1

The mean item complexity is 1.5, suggesting that most items load on one factor more than another, which is ideal for a simple structure. Looking at the model fit statistics in Table 9, an RMSR of 0.04 indicates a good fit whereas lower values (generally less than 0.08) indicate a better fit. For the fit-based off-diagonal values, the value of 1 suggests an excellent model fit. Finally, the correlation of factor scores with factors indicate that the factor scores are closely related to the underlying factors with values of 0.94 for ML2 and 0.99 for ML1.

#### Exploratory Factor Analysis (Performance)

For the second section, a polychoric correlation matrix was also performed for the EFA, but this time the binary items (P1 and P4) were removed, and the 3-point Likert scale items were examined after reversing the only negative scored item of this section (P5) that was replaced by P5_rev. The output matrix (Table 10) contains polychoric correlation coefficients that indicate the strength and direction of the association between each pair of items. The results here show a moderate to strong correlation between the tested items indicating that they are measuring similar constructs, suggesting good reliability.

**Table 10:** Polychoric correlation results for the items of performance

	P2	P3	P5_rev	P6	P7	P8	P9
P2	1.000	0.725	0.578	0.651	0.547	0.587	0.706
P3		1.000	0.549	0.612	0.535	0.567	0.687
P5_rev			1.000	0.496	0.471	0.583	0.692
P6				1.000	0.551	0.621	0.733
P7					1.000	0.825	0.792
P8						1.000	0.842
P9							1.000

The item-total correlation for P1 and P4 are 0.363 and 0.324, respectively. These results suggest a moderate positive relationship with the total score. In other words, as the scores on P1 or P4 increase, there will be a corresponding increase in the total score. While these correlations are significant, they are not particularly high, which may suggest that each of P1 and P4 has a moderate consistency with the overall construct.

#### Confirmatory Factor Analysis

Confirmatory Factor Analysis (CFA) was conducted to validate the structure of the awareness model for household disinfectant use. The CFA model employed the DWLS estimator with NLMINB optimization, selected for robustness with ordinal data.

##### Section 1 (Awareness)

The model fit statistics were evaluated using multiple indices, starting with the Chi-Square test that yielded a statistic of 146.937 with 27 df and a *P*-value of 0.000, indicating a lack of perfect fit, which is often expected in CFA due to sensitivity to sample size (Table 11). The CFI presented a value of 0.990 that exceeds the 0.95 threshold, signifying a strong fit. Also, the Tucker-Lewis Index (TLI) showed a value of 0.986, which further supports a good fit, aligning with the recommended standard. Additionally, the RMSEA of 0.077 falls within the acceptable range (Below 0.08), suggesting a reasonable model fit. Similarly, the SRMR, with a value of 0.056, meets the good-fit criterion (Below 0.08), reinforcing overall model suitability.

**Table 11:** CFA output for the items of awareness

Chi-Square Test	Test Statistic	146.937
	*P*-Value	0.000
	df	27
Model Fit Indices	CFI	0.990
	TLI	0.986
	RMSEA	0.077
	SRMR	0.056

To assess the quality and the strength of the measurement model and to ensure that the latent constructs are accurately represented by the observed items, several estimates were performed (Appendix B). For parameter estimates, all items (A1 to A9) showed significant loadings on the latent variable for awareness (*P* < 0.001). Standardized factor loadings ranged from 0.667 (A2) to 0.828 (A1), with A1 as the reference indicator. A1 and A4, with loadings of 0.828 and 0.818 respectively, emerged as the most influential items, while A2, with the lowest loading (0.667), contributed less prominently. These loadings underscore the relative importance of each item in defining the awareness construct.

For threshold estimates, all items were significant (*P* < 0.001), validating the consistency and appropriateness of response categories across the Likert-scale items. Finally, for the variance estimates, the latent variable for awareness accounted for 0.686 of the variances in the observed indicators, demonstrating its substantial explanatory power.

##### Section 2 (Performance)

The model fit output presented in Table 12 was assessed using multiple indices, beginning with the Chi-Square test, which produced a statistic of 254.392 with 27 df and a *P*-value of 0.000, indicating an imperfect fit—a common outcome in CFA due to sample size sensitivity. The CFI scored 0.975, exceeding the 0.95 threshold and indicating a strong fit. Similarly, the TLI was 0.967, reinforcing the model's adequacy by meeting recommended standards. The RMSEA was 0.090, with a 90% confidence interval from 0.094 to 0.118, slightly above the preferred cutoff of 0.08 but within the acceptable range for preliminary or exploratory models. Finally, the SRMR value was 0.083, close to the typical acceptability threshold of 0.08, suggesting minor areas for model improvement but generally indicating adequate fit.

**Table 12:** CFA for the items of performance

Chi-Square test	Test Statistic	254.392
	*P*-Value	0.000
	df	27
Model Fit Indices	CFI	0.975
	TLI	0.967
	RMSEA	0.090
	SRMR	0.083

Overall, the fit indices indicate that the model is reasonably appropriate for capturing the underlying structure of the "Performance" latent variable, though there may be minor areas where the fit could be improved.

The latent variable "Performance" was effectively measured (Appendix C), as shown by significant item loadings ranging from 0.758 (P5_rev) to 0.924 (P1), with high loadings on items P1, P4, and P9, indicating their strong contribution to the construct. Each item's high statistically significant loading (*P* < 0.001) confirms consistent alignment with the underlying "Performance" factor. Threshold estimates further illustrate response patterns, such as P2’s threshold at −1.405, where participants tended to select lower levels on the Likert scale, highlighting the distribution shift across categories. The latent variable variance of 0.853 (*P* < 0.001) reinforces the construct's presence and the diversity in responses, supporting "Performance" as a well-defined, variable factor within this sample.

To investigate the dissatisfactory results of the RMSEA and SRMR, the confirmatory analysis was repeated for the 3-point Likert scale items (excluding the binary items P1 and P4). The results are presented in Table 13. All the calculated parameters (*P*-value of χ^2^ < 0.001, CFI > 0.9, TLI > 0.9, and each of RMSEA and SRMR was < 0.08) were good including the RMSEA and the SRMR. This justifies the unacceptable values of RMSEA and SRMR in the previous test as the binary items were responsible for this discrepancy due to the nature of the different scales used in one test.

**Table 13:** CFA for the items of performance without the binary items

Chi-Square test	Test Statistic	138.599
	*P*-Value	0.000
	df	14
Model Fit Indices	CFI	0.988
	TLI	0.983
	RMSEA	0.049
	SRMR	0.059

All factors’ loadings are statistically significant (*P* < 0.001), confirming that each item significantly contributes to the latent construct of Likert items. Additionally, the variances for each item and the latent variable are statistically significant (*P* < 0.001), suggesting that the items exhibit unique variability in addition to the common variance explained by the latent factor (Appendix D).

## Discussion

The results support the instrument's effectiveness, with statistically significant loadings for all items, indicating strong alignment with the underlying construct. Each item’s unique variance further underscores the questionnaire's ability to capture diverse aspects of household awareness and practices. The following sections interpret these findings in the context of existing literature, address study limitations, and suggest implications for future research and public health initiatives.

### Face validity

Based on the rating of the nine experts, the result of face validity indicates that for all of the seven parameters (difficulty, obscurity, level of importance, simplicity, clarity, test of appropriateness, and level of relevance) and for both domains awareness and performance, the ratings generally ranged between 0.90 and 1.00 except for simplicity with the awareness domain (0.86–0.97) and test of appropriateness for performance (0.875–0.97); however, the average face validity for the whole questionnaire was 0.97. This high face validity rating aligns with best practices for scale development, reflecting the relevance of the questionnaire [42].

### Content validity

The CVR values for the seven markers for the two domains awareness and performance are presented in Table 2. For the first six items, the CVR values are reasonably high, but, for items 7, 8, and 9, the CVR for the most is considered low. However, the average CVR for the whole questionnaire was 0.81, indicating a good CVR. As used in several studies, both the face and the content validity contribute to the refinement of the questionnaire through experts’ evaluations [33, 42, 43].

### Construct validity

#### Reliability testing

In this study, the Cronbach’s alpha values of 0.854 for Sect. 1 (Awareness) and 0.883 for Sect. 2 (Performance) indicate that the questionnaire items exhibit strong internal consistency, signifying that the items within each section reliably measure the intended constructs [44]. Typically, a Cronbach’s alpha value above 0.70 is considered acceptable, with values above 0.80 reflecting good reliability. Therefore, these results suggest that both sections of the questionnaire are reliable for assessing knowledge and practices related to household cleaning disinfectants. High internal consistency also implies that the items within each section are likely aligned with their respective constructs, bolstering the validity of the questionnaire.

#### Principal Factor Analysis (For both sections)

The high KMO values for awareness (0.879) and the performance items (0.891) and significant Bartlett’s test suggest that the data structure in each of the awareness and performance sections are appropriate for factor analysis, with items interrelated enough to form a coherent factor structure [45]. The communalities demonstrate that most items are moderately explained by the extracted components, supporting their relevance in measuring awareness and performance. The total variance explained by the first two components (58.3%) and (64.7%) highlights that awareness and performance about household product safety can each be largely captured by one dominant factor, with a secondary factor contributing a smaller portion. This indicates that awareness may be mostly unidimensional with some additional aspects, justifying a focus on the first component for further interpretation.

#### Exploratory Factor Analysis (For Sect. 1)

The EFA results for the first section indicate a well-structured model with two key factors explaining a substantial portion of the variance in respondents' awareness of household cleaning disinfectants. Factor 1 (ML2) and Factor 2 (ML1) account for 41% and 24% of the total variance, respectively, yielding a cumulative variance of 65%. This indicates that these two factors capture a significant part of the variation in responses [46]. The mean item complexity of 1.5 suggests that most items predominantly load onto one factor, supporting a simple structure that aligns with our expectations for distinct underlying constructs. Model fit statistics reinforce the adequacy of this structure, with an RMSR of 0.04 indicating an excellent fit, as values below 0.08 generally reflect good fit quality. Additionally, the high correlation of factor scores with the factors (0.99 for ML1 and 0.94 for ML2) demonstrates that the scores closely represent the underlying dimensions identified. These results suggest that the factors identified in this section are both meaningful and well-defined, providing a reliable basis for further interpretation of participants' awareness levels.

Given the results obtained, the first section of the questionnaire is reliable and valid, effectively capturing the intended constructs associated with safe cleaning and disinfection practices.

#### Exploratory Factor Analysis (For Sect. 2)

The EFA for the performance section, conducted using a polychoric correlation matrix, demonstrated moderate to strong correlations among the 3-point Likert items (P2, P3, P5_rev, P6, P7, P8, and P9). This suggests that these items consistently capture related aspects of the underlying construct, indicating good reliability in this section [47]. The reversed item (P5_rev) also aligned well with the other items, confirming that reverse scoring was handled correctly.

The binary items (P1 and P4) were evaluated separately through item-total correlation, revealing moderate positive relationships (0.363 for P1 and 0.324 for P4) with the total score. While these correlations indicate a consistent association between these items and the overall construct, their moderate values suggest a lesser alignment compared to the Likert items. These findings imply that, although P1 and P4 contribute meaningfully to the overall performance measure, they capture distinct elements of the construct relative to the Likert-scale items, providing a more nuanced view of performance within this section.

#### Confirmatory Factor Analysis (Awareness)

The model fit indices (CFI, TLI, RMSEA, and SRMR) collectively affirm that the structure effectively represents the data. The significant factor loadings indicate that each indicator meaningfully contributes to the awareness construct, with items A1 and A4 being especially strong contributors. This suggests a solid understanding of household disinfectant knowledge among respondents. Overall, the findings confirm the relevance of the knowledge construct in gauging awareness of cleaning practices [48].

#### Confirmatory Factor Analysis (Performance)

The findings confirm that the model effectively captures the "Practices of Household Cleaning Disinfectant" construct, with strong factor loadings across items and generally acceptable model fit indices. High standardized loadings for items P1, P4, and P9 indicate their effectiveness in assessing the construct, potentially highlighting key aspects of household cleaning practices. The solid factor structure shows that this section of the questionnaire reliably reflects participants' disinfectant practices. However, the RMSEA and SRMR values suggest minor misfits, which could be improved by refining item wording or adding factors in future evaluations. Overall, these results indicate that the questionnaire serves as a reliable tool for assessing disinfectant practices, with slight adjustments potentially enhancing its accuracy.

## Implications for practice

The HDCQ is a tested Household Disinfectants-Cleaners Questionnaire that has several potential applications to inform public health intervention, future studies, and policy development:

### Future research and cross-cultural adaptation

The HDCQ provides a standardized measuring instrument for assessing public knowledge and practice on household disinfectants. Future studies can utilize the questionnaire in diverse geographic and cultural settings to assess knowledge and behavior patterns. Cross-cultural validation of the HDCQ would then further limit its use and validity across various populations.

### Public health interventions

Findings can be employed to create focused education campaigns to upgrade consumer literacy and guarantee safe disinfectant usage. Consequently, public health agencies are able to use the HDCQ to establish awareness of deficiencies and unsafe habits and allow interventions to target specific misconceptions and behavior risks. Additionally, the HDCQ can also be employed as a pre-and post-intervention measure to evaluate the effectiveness of awareness campaigns.

### Policy and regulatory applications

Policymakers can incorporate the HDCQ into national health surveillance programs to monitor trends in disinfectant use and measure public compliance with safety standards. In the same manner, regulatory agencies can utilize the findings to set more transparent labeling requirements, consumer safety standards, and risk communication plans for household cleaning products. The HDCQ may be used as a reference point for contrasting current awareness initiatives and informing data-driven policy changes in the future.

By placing emphasis on these practical implications, this study provides a significant input to public health, scholarship, and policymaking practice for the maintenance of safe and informed household disinfectant use.

### Limitations

In the "Performance" section of the questionnaire, nine items were included, with seven items utilizing a 3-point Likert scale and two items structured as binary responses. This mixed item format presented challenges in the validation process, requiring a tailored approach for both exploratory and confirmatory analyses. Specifically, traditional tetrachoric correlation was not suitable for the binary items due to their limited number. Therefore, in the exploratory analysis, a total-item correlation approach was applied to assess the relationship between each item and the overall scale, providing a more accurate assessment of internal consistency across both item types. For confirmatory analysis, adjustments were also made to account for the mixed-format nature, ensuring that both Likert-scale and binary items were appropriately integrated in the model.

This study utilized a convenience sampling method, which may introduce biases due to self-selection and sample homogeneity, limiting the generalizability of the findings. However, the authors ensured that the sample included acceptable diversity in age groups and gender, which may help reduce potential biases. Future research could benefit from probability-based sampling to improve representativeness.

## Conclusions

Following extensive validation steps, including face, content, construct, and reliability analyses, the HDCQ tool demonstrates robust psychometric properties, confirming it as a valid and reliable tool. The face and content validity assessments, based on expert evaluations, underscored the questionnaire’s relevance and appropriateness for measuring household awareness and performance related to household disinfectants-cleaners. High internal consistency and significant factor loading in both sections further affirm that the items reliably capture the intended constructs.

The exploratory and confirmatory factor analyses reveal a clear factor structure, providing strong support for the instrument’s capacity to measure awareness and performance constructs accurately. Although some minor model fit improvements were suggested, these do not detract from the instrument’s overall reliability and validity, highlighting its applicability for future research and practical interventions in public health.

In summary, this validated HDCQ tool offers a sound instrument for assessing household cleaning and disinfection practices, supporting its use in varied research settings and as a foundation for initiatives aiming to improve public health and safety in domestic environments.

## Supplementary Information

Supplementary Material 1.

## Data Availability

The datasets used and/or analyzed during the current study are available from the corresponding authors on reasonable request. Additional information is included in the Appendices.

## Abbreviations

UAE: United Arab Emirates
PFA: Principal Factor Analysis
KMO: Kaiser–Meyer–Olkin
EFA: Exploratory Factor Analysis
CFA: Confirmatory Factor Analysis
CVR: Content Validity Ratio
COVID-19: Coronavirus Disease 2019
SARS-CoV-2: Severe Acute Respiratory Syndrome Coronavirus-2
CDC: Centers for Disease Control and Prevention
SDG: Sustainable Development Goal
HDCQ: Household Disinfectants-Cleaners Questionnaire
IRB: Institutional Review Board
I-CVI: Item level Content Validity Index
UA: Universal Agreement
SPSS: Statistical Package for Social Sciences
DWLS: Diagonally Weighted Least Squares
(NLMIN)B: Non-Linear MINimization using Bounds
CFI: Comparative Fit Index
TLI: Tucker Lewis Index
RMSEA: Root Mean Square Error of Approximation
SRMR: Standardized Root Mean Square Residual
AWR: Awareness
PERF: Performance
Df: Degrees of freedom
