# Supplementary material for: Validity and reliability of Household Disinfectants-Cleaners Questionnaire (HDCQ) to investigate public awareness and performance in the Emirate of Abu Dhabi
Source: BMC Public Health. 2025 Mar 29;25:1201. doi: 10.1186/s12889-025-22317-y (PMC11955105; doi:10.1186/s12889-025-22317-y)

**Appendices**

**Appendix A**

**Table S1.** Inter-item correlation matrix for awareness

|  | A1 | A2 | A3 | A4 | A5 | A6 | A7_rev | A8_rev | A9 |
| --- | --- | --- | --- | --- | --- | --- | --- | --- | --- |
| A1 | 1.000 | 0.469 | 0.483 | 0.498 | 0.517 | 0.400 | 0.429 | 0.353 | 0.451 |
| A2 |  | 1.000 | 0.310 | 0.324 | 0.332 | 0.346 | 0.335 | 0.308 | 0.340 |
| A3 |  |  | 1.000 | 0.558 | 0.497 | 0.394 | 0.310 | 0.405 | 0.439 |
| A4 |  |  |  | 1.000 | 0.510 | 0.483 | 0.354 | 0.348 | 0.403 |
| A5 |  |  |  |  | 1.000 | 0.459 | 0.316 | 0.326 | 0.393 |
| A6 |  |  |  |  |  | 1.000 | 0.318 | 0.315 | 0.508 |
| A7_rev |  |  |  |  |  |  | 1.000 | 0.581 | 0.418 |
| A8_rev |  |  |  |  |  |  |  | 1.000 | 0.391 |
| A9 |  |  |  |  |  |  |  |  | 1.000 |

**Table S2.** Inter-item correlation matrix for performance

|  | P1 | P2 | P3 | P4 | P5_rev | P6 | P7 | P8 | P9 |
| --- | --- | --- | --- | --- | --- | --- | --- | --- | --- |
| P1 | 1.000 | 0.521 | 0.468 | 0.319 | 0.529 | 0.492 | 0.496 | 0.512 | 0.582 |
| P2 |  | 1.000 | 0.582 | 0.501 | 0.447 | 0.502 | 0.391 | 0.412 | 0.533 |
| P3 |  |  | 1.000 | 0.550 | 0.429 | 0.475 | 0.383 | 0.406 | 0.512 |
| P4 |  |  |  | 1.000 | 0.535 | 0.479 | 0.401 | 0.428 | 0.527 |
| P5_rev |  |  |  |  | 1.000 | 0.389 | 0.342 | 0.421 | 0.512 |
| P6 |  |  |  |  |  | 1.000 | 0.399 | 0.447 | 0.533 |
| P7 |  |  |  |  |  |  | 1.000 | 0.662 | 0.618 |
| P8 |  |  |  |  |  |  |  | 1.000 | 0.681 |
| P9 |  |  |  |  |  |  |  |  | 1.000 |

**Table S3**. Total variance explained for the items of awareness

| Component | **Initial Eigenvalues** | | |
| --- | --- | --- | --- |
|  | Total | % of Variance | Cumulative % |
| 1 | 4.266 | 47.396 | 47.396 |
| 2 | 0.980 | 10.890 | 58.287 |
| 3 | 0770 | 8.560 | 66.847 |
| 4 | 0.702 | 7.803 | 74.650 |
| 5 | 0.538 | 5.980 | 80.629 |
| 6 | 0.526 | 5.844 | 86.473 |
| 7 | 0.482 | 5.351 | 91.825 |
| 8 | 0.382 | 4.248 | 96.073 |
| 9 | 0.353 | 3.927 | 100.000 |

**Table S4.** Total variance explained for items of performance

| Component | **Initial Eigenvalues** | | |
| --- | --- | --- | --- |
|  | Total | % of Variance | Cumulative % |
| 1 | 4.884 | 54.269 | 54.269 |
| 2 | 0.942 | 10.464 | 64.732 |
| 3 | 0.659 | 7.319 | 72.052 |
| 4 | 0.649 | 7.208 | 79.259 |
| 5 | 0.533 | 5.924 | 85.183 |
| 6 | 0.407 | 4.527 | 89.710 |
| 7 | 0.353 | 3.925 | 93.635 |
| 8 | 0.297 | 3.303 | 96.938 |
| 9 | 0.276 | 3.062 | 100.000 |

**Appendix B**


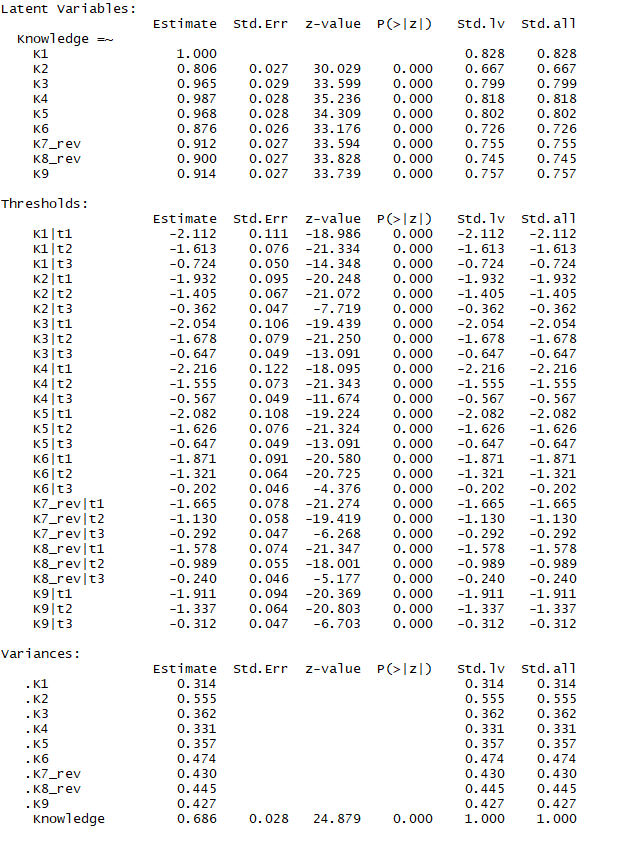


**Appendix C**


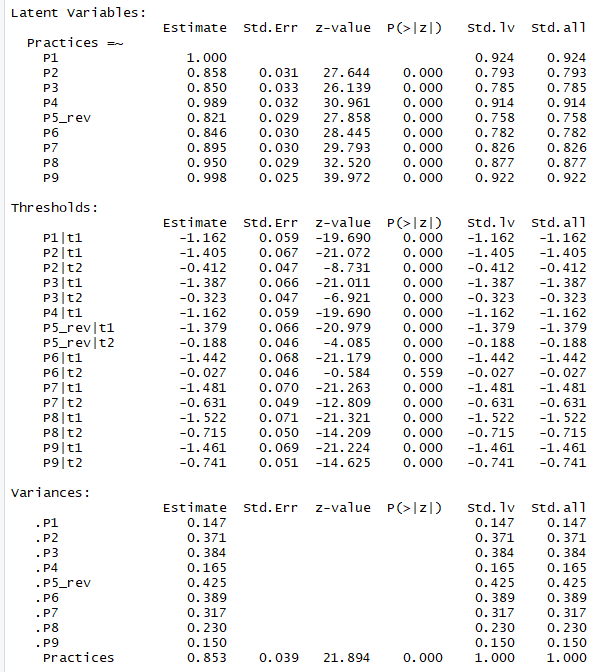


**Appendix D**


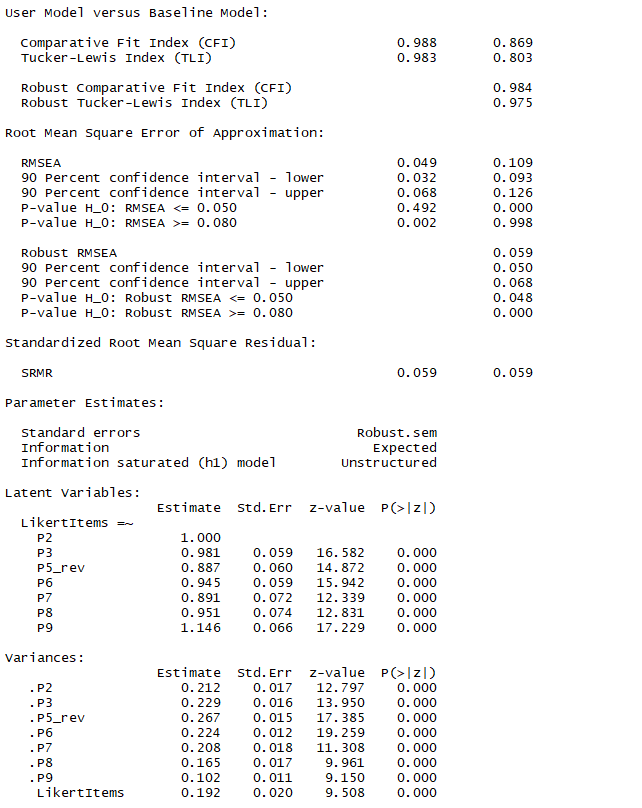

Supplement: Supplementary file 1 — Supplementary Material 1. [file 12889_2025_22317_MOESM1_ESM.docx]
